# A review of auditory processing and cognitive change during normal ageing, and the implications for setting hearing aids for older adults

**DOI:** 10.3389/fneur.2023.1122420

**Published:** 2023-06-20

**Authors:** Richard Windle, Harvey Dillon, Antje Heinrich

**Affiliations:** ^1^Audiology Department, Royal Berkshire NHS Foundation Trust, Reading, United Kingdom; ^2^NIHR Manchester Biomedical Research Centre, Manchester, United Kingdom; ^3^Department of Linguistics, Macquarie University, North Ryde, NSW, Australia; ^4^Division of Human Communication, Development and Hearing, School of Health Sciences, University of Manchester, Manchester, United Kingdom

**Keywords:** ageing, cognitive performance, hearing aids, auditory processing, compression speed, compression ratio, noise reduction

## Abstract

Throughout our adult lives there is a decline in peripheral hearing, auditory processing and elements of cognition that support listening ability. Audiometry provides no information about the status of auditory processing and cognition, and older adults often struggle with complex listening situations, such as speech in noise perception, even if their peripheral hearing appears normal. Hearing aids can address some aspects of peripheral hearing impairment and improve signal-to-noise ratios. However, they cannot directly enhance central processes and may introduce distortion to sound that might act to undermine listening ability. This review paper highlights the need to consider the distortion introduced by hearing aids, specifically when considering normally-ageing older adults. We focus on patients with age-related hearing loss because they represent the vast majority of the population attending audiology clinics. We believe that it is important to recognize that the combination of peripheral and central, auditory and cognitive decline make older adults some of the most complex patients seen in audiology services, so they should not be treated as “standard” despite the high prevalence of age-related hearing loss. We argue that a primary concern should be to avoid hearing aid settings that introduce distortion to speech envelope cues, which is not a new concept. The primary cause of distortion is the speed and range of change to hearing aid amplification (i.e., compression). We argue that slow-acting compression should be considered as a default for some users and that other advanced features should be reconsidered as they may also introduce distortion that some users may not be able to tolerate. We discuss how this can be incorporated into a pragmatic approach to hearing aid fitting that does not require increased loading on audiology services.

## 1. Introduction

In this review paper, we highlight the importance of recognizing the effects of normal ageing on hearing aid fitting because it has a considerable impact on an individual’s understanding of speech and ability to benefit from hearing aids. Auditory processing and cognition decline throughout early, middle and late adult life and undermine our ability to hear in all types of listening situations. “Normal ageing” refers to non-pathological changes in cognition and auditory ability with age (1). We do not discuss pathological changes with age such as mild cognitive impairment or dementia. Whilst considerable research efforts continue to be deployed to understand the association between hearing loss and dementia, we believe it is particularly important to address normally-ageing older adults because they represent the vast majority of patients attending audiology clinics. In the UK National Health Service (NHS), older adults (over 55 years of age) represent 78% of adult audiology attendances and 87% of those fitted with hearing aids (2); and reported hearing problems appear more strongly associated with age than the degree of peripheral hearing loss, which suggests that processes other than peripheral hearing play a role (2, 3). For the purposes of this review we define “peripheral” changes as those restricted to the outer, middle and inner ear and auditory nerve. We define “central” changes as those within the central auditory nervous system, from the level of the cochlear nucleus up to the cortex, which includes elements of cognition. The aim of this review is to determine the relevant factors in auditory processing and cognition that might have implications for the way in which we set hearing aids, and to define the best approach to setting hearing aids for normally-ageing older adults based on the evidence in the literature. Whilst we do not discuss the relationship between hearing and dementia, it is likely that any recommendations regarding setting hearing aids for those with reduced degrees of cognition due to normal ageing will be particularly applicable to those with pathological cognitive decline.

We first provide a brief review of age-related change to central auditory processing and how this alters dependence on different elements of speech (Section 2). We then offer a short review of the basic concepts of cognitive change with age (Section 3), and how a decline in some elements of cognition also alters speech perception, particularly undermining listening in more challenging circumstances (Section 4). This understanding then enables us to determine how hearing aid parameters and fitting procedures might affect older adults’ listening ability. In Section 5, we derive a set of evidence-based principles for fitting hearing aids to older adults, and assess specific hearing aid parameters against these principles. We review evidence for an association between some elements of cognition and the benefit, or increased impairment, that may be caused by hearing aid processing strategies. We particularly focus on evidence that addresses the benefit, or harm, caused by fast or slow-acting compression speeds for those with different degrees of cognition. We also assess why the evidence may not always appear consistent. We then offer a discussion of how one might determine practical fitting guidelines for audiologists who predominantly see older adults in clinic (Section 6). Finally, we discuss gaps in the current evidence-base and what further research might be needed (Section 7).

## 2. The effects of ageing on auditory processing

The central auditory nervous system (referred to simply as the “auditory system” in subsequent text) is a uniquely complex sensory network, engaging peripheral sensory organs, multiple processing layers in the brainstem, the auditory cortex and wider cortex, culminating in conscious perception and understanding, as well as extensive “top-down” control. The processing of different aspects of speech is distributed across the auditory system. For example, some neurons are tuned to overall amplitude modulation from lower to higher levels of the system (4) and others are phase-locked to periodic patterns in the acoustic detail of speech (5, 6). Segregating and grouping sounds depends on multiple binaural processes from the cochlear nucleus up to the cortex, dependent on effective neural synchrony (7). Multiple pathways in the cortex, largely between temporal and frontal lobes, support a hierarchical process of word recognition, integration into phrases and sentences, syntax (grammar) and semantics (meaning) (8–10). Higher-level processes also depend on the relevance of sounds and lower-level processes can be enhanced via top-down control, enabling selective processing (11).

Age-related changes to the underlying neural infrastructure cause progressive “central” auditory deficits with increasing age (1, 12–17). As “lower” and “higher” level processes are interrelated, the effects of peripheral and central decline can be difficult to dissociate. Age-related cochlear damage generates a degraded input to the auditory system. The degraded nature of the input is exacerbated by the loss of synapses and auditory nerve fibers, often referred to as “hidden hearing loss.” This synaptic loss particularly affects fibers that are sensitive to louder sounds and may particularly impair speech-in-noise (SIN) perception (18–20). With increasing age there is a general reduction in the density of connections in the brainstem and cortical structures involved in auditory processing (14, 21). A deterioration in brainstem structures and the cortex impairs timing information, gap detection, localization, spectral processing and efferent control of the hair cells (22–25). These combined changes can undermine auditory scene analysis, impair an individual’s ability to attend to a target speaker (26) and reduce the ability to distinguish elements of speech in quiet (SIQ), non-familiar accents and SIN (27, 28).

Temporal information in speech cues can be differentiated across a number of frequency bands (29): information about the speech envelope (ENV) is carried primarily in the frequency band below 50Hz; information about voicing and periodicity primarily in the frequency band between 50 and 500Hz; and information about temporal fine structure (TFS) in the frequency band above 500Hz. ENV and TFS are the most important cues forming the basis of speech perception, frequency perception and localization (30). However, for those with sufficient hearing, ENV and TFS provide redundant cues that support processing of the speech signal (31). A difference in the pitch of voicing helps differentiate individuals in multi-talker situations (32). Vocoder experiments that replaced TFS with a carrier signal or noise, which was modulated by ENV, demonstrated that only a small number of frequency bands are required for successful speech perception in quiet even when TFS was lacking (33). However, the same was not true when speech was presented in a noisy background. Adding back TFS to a signal progressively improved SIN performance (34, 35). The ability to make use of ENV with little TFS can also be evidenced by the success of cochlear implants, which provide a grossly reduced representation of spectral detail (29, 36). Besides supporting speech perception in noise (36–38), TFS also supports binaural localization and separation of competing sounds (39), perception of pitch and music (29, 30) and perception of motion (40). Individuals with peripheral hearing impairment cannot make good use of TFS which can lead to difficulties hearing in noisy situations (41).

Comparisons between young and old listeners with matched normal hearing, intelligence and education suggest that speech perception declines with age, demonstrating a greater effect than can be predicted by hearing thresholds (3, 42–44). Monaural TFS sensitivity declines constantly throughout early-to-late adulthood (45), whereas monaural processing of ENV is less affected (46, 47). Binaural processing of both TFS and ENV declines with age, although declining TFS sensitivity has some association with the degree of hearing loss whereas the association between ENV sensitivity and hearing loss is weak (46, 47). Besides TFS, cognitive performance has also been found to predict speech perception. Although both TFS sensitivity and some cognitive domains decline with age, they do not appear to be directly related to each other once the effect of age is removed (48). TFS sensitivity was found to be a stronger factor than cognitive ability and ENV sensitivity in predicting speech perception in quiet for normal hearing listeners, and cognition the stronger factor for predicting performance in noise (3). Reduced SIN perception with increasing age can be more generally associated with reduced cognitive abilities (49), reduced coding accuracy and TFS sensitivity (50, 51), poorer binaural processing (52) and sound segregation (26). Sensitivity to periodicity perception also declines with age, affecting the perception of intonation (53), discrimination of voicing (54) and the emotional content of speech (55, 56). Some elements of speech perception are therefore specific to auditory processing, whereas others are dependent on domains of cognition not specific to auditory processing.

## 3. Cognitive change during normal ageing

“Cognition” refers to the processes of acquiring, retaining and using knowledge (57) and has many constituent elements, including reasoning, memory, speed, knowledge, reading, writing, maths, sensory, and motor abilities (58). One can differentiate cognition from auditory processing because the latter is specific to the analysis of sound, whereas the former is not. Whilst the auditory system processes sound, cognitive processes develop meaning and understanding from the sound. However, it should be recognized that this is a simplistic description because of the inter-related nature of the systems which together deploy “bottom-up” and “top-down” processes. Some elements of cognition decline with age, but some remain stable or improve (59). Cognitive abilities can be separated into two basic domains (60, 61): “fluid intelligence,” characterized as the speed and ability to resolve problems in novel situations; and “crystallized intelligence” that is an accumulation and use of skills, knowledge and experience. Fluid intelligence generally peaks at about 20 years of age and declines at a consistent rate throughout adult life, whereas crystallized intelligence increases with age. As a result, an individual’s overall performance in psychometric tests of intelligence remains largely stable through most of adult life (60, 62). It should be noted that the range of ability between individuals also increases with age (21, 60, 63) so age alone is not a good measure of specific elements of cognitive function. Individuals of different ages may therefore perform equally well at a complex task, but may be using a different blend of cognitive abilities to complete the task based on the strength of different cognitive domains (64). Accordingly, an overall measure of “general cognition” is not a useful concept when considering a specific complex task, such as listening. Cognitive reserve, developed through education and other lifetime experiences, appears to provide some individuals with greater resilience against cognitive decline (65). This, and other genetic, environmental, health and lifestyle factors, will mediate any cognitive change with age (63) and an individual might further modify function via cognitive training and physical exercise (66). In short, it is important to determine the specific elements of cognition that relate to different tasks rather than make any assumption of homogeneity amongst an age group.

Several theories have been proposed to describe the process of decline in some cognitive abilities. The processing-speed theory (67) suggests that a decline in speed results in processes failing to complete in time to be useful, and that slower processes reduce the amount of available processing capacity. A reduction in processing capacity, caused either by a reduction in processing speed or by other processes of decline, could undermine episodic memory (recollection of personal experiences) and the initiation of more complex processes (68). Note that episodic memory declines more with age compared to semantic memory (recollection of facts) and procedural memory (unconscious performance of tasks) (59). A second theory suggests that there is a limit on the amount of processing resources (69). Based on this idea, Baddeley & Hitch (70) introduced the concept of “working memory” (WM) as a system that stores and processes information relevant to a current task. A third theory postulates that an age-related failure to filter out irrelevant information is the cause for WM to become “cluttered,” thus reducing available capacity (71). Each of the individual theories cannot explain all of the features of cognitive decline and it is likely that there are multiple processes with cumulative effects (59).

**Figure 1:**
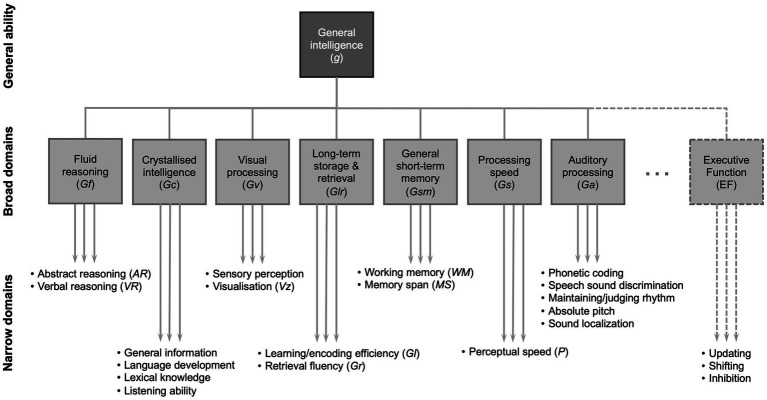
A partial representation of the Cattell-Horn-Carroll (CHC) theory of cognitive abilities showing general intelligence (*g*) consisting of broad domains of cognitive function, under which there are narrow domains that can be assessed with specific tests. Figure developed from (72, 73). Note that there are a greater number of broad domains and the narrow domains shown are only selected examples of those in each broad domain (24).

One way to categorize the basic concepts of fluid and crystallized intelligence into further subtypes of cognition, particularly useful in relating specific processes to speech perception, is provided by the Cattell-Horn-Carroll model (72, 73) (Figure 1). The model describes a three-level hierarchy of cognitive abilities: general ability, broad domains and narrow domains. Broad domains are basic characteristics that manage a range of behaviors. Narrow domains are highly specialized and may be specific to certain types of task. Auditory processing (Ga) is included as a broad domain in the CHC model (Figure 1). Some elements of overall sound processing and perception are specific to Ga, whereas some elements rely on other narrow domains. Note that the generalized concept of fluid intelligence is not specifically shown in the model, but includes most of the broad domains of the model, i.e., those other than crystallized intelligence (Gc). Nevertheless, in general terms, abilities in the wide domain of fluid intelligence are those that tend to decline with increasing age.

Note that ongoing refinements to the model introduce further broad and narrow domains beyond those shown here (72). Episodic memory is contained within the definition of learning/encoding efficiency (Gl) in the CHC model (Figure 1) (73). Executive function is not commonly included in the Cattell-Horn-Carroll model and there is no clear consensus on its definition (73). It may be thought of as a cognitive “control” function that mediates narrow domain abilities, although its three core elements may be considered as separate domains that: enable “shifting” between tasks; monitoring tasks and “updating” WM as necessary; and “inhibiting” other tasks or automated responses in order to complete a task (74).

## 4. The effects of cognitive change on speech perception

In this section we focus on the elements of cognition that may underlie impaired listening ability. Not enough is currently known about how specific narrow domains of cognitive ability (Figure 1) relate to auditory performance in specific listening situations. However we do know that WM often plays a role and is frequently associated with SIN performance (75, 76), understanding fast speech (77) or with general auditory performance across a range of tasks (78, 79). WM is more predictive of SIN performance in older age groups compared to younger groups (80) or in more challenging conditions, such as a lower signal-to-noise ratio (81). Older adults with greater WM resources may be better able to adapt to difficult listening situations (78, 82). WM is often used as a broad term and encompasses both storage and processing-dependent WM tasks. These different types of tasks can have different associations to different types of listening situations (75, 83–85). Executive function is another cognitive factor that is often associated with listening and the degree of listening effort (86). Inhibition, which a sub-domain of executive function (Figure 1), and processing speed are also cognitive functions associated with reduced auditory performance (87–89). Different domains of cognition appear to be more strongly predictive of listening ability for more difficult listening tasks (90, 91). A meta-analysis (92) provided an overview over various cognitive functions and their association with different SIN tasks. It showed that SIN performance correlated with measures of processing speed (*r* = 0.39), inhibition (r = 0.34), WM (*r* = 0.28), and episodic memory (*r* = 0.26). These are all narrow cognitive domains that deteriorate throughout adulthood (62, 67, 68, 71, 93) and their effects may be, in part, additive.

One consequence of the changes to hearing and cognition may be an increase in effort when listening (94, 95). Listening effort is defined in the Framework for Understanding Effortful Listening (FUEL) (96) as an allocation of cognitive resources to overcome listening obstacles. It is a finite resource and its capacity will be expended at different rates dependent on the inherent difficulty of a task and an individual’s motivation to overcome obstacles in listening. Listening effort likely engages multiple neurological systems (97) and is an important factor in speech perception. It should be considered in the context of age-related cognitive decline because it may also affect the motivation of individuals to comply with treatment or engage socially (98, 99). In addition, hearing aids have the potential to reduce, or increase, listening effort (100–104).

The relative importance of hearing loss, auditory processing and cognitive function for predicting overall auditory performance will depend on the specific listening situations encountered (76), which will vary in real-life (105). Different forms of SIN test engage different narrow cognitive domains (92). SIN tasks can be distinguished according to the target and masker signal, and both of these affect the type of processing needed for successful listening (92). The type of masking noise has a considerable effect; individuals perform better in steady-state noise compared to multi-talker babble and the latter yields stronger associations with cognitive function (85, 92, 106–108). An intelligible masking signal provides both energetic and informational masking, and is most likely to divide the attention of a listener, offering the greatest cognitive challenge (75), although other cues such as the different gender or fundamental frequency of the signal and noise source can overcome some effects (109).

A number of speech models have attempted to conceptualize the role of different cognitive functions within the pathway of speech understanding. Gordon-Salant et al. (21) adapted a theoretical model (110) to describe a bottom-up process of hierarchical signal processing. The “sensory system” undertakes spectral and temporal processing to discriminate between sound sources. This is further refined by the “perceptual system,” employing inhibition to direct attention, and the “cognitive system” that analyses and identifies words, and develops meaning, feeding back to lower levels of the system, requiring processing speed, WM capacity and semantic knowledge, also engaging long-term memory (Glr). The model by Bronkhorst (11) is broadly similar in principle and highlights the role of attention control, an element of executive function, in which attention is triggered by certain signal characteristics, that then engenders selective processing at lower levels, or “pre-attentive” stages, of the auditory system. The Ease of Language Understanding (ELU) model (111, 112) largely focusses on situations in which there is conflict between an input signal and lexical information stored in memory, e.g., due to an unusual accent, which then requires engagement of a feedback loop to resolve it, particularly requiring WM, executive function and learning/encoding efficiency. Taken as a whole, these models highlight the critical dependence of listening on certain specific narrow cognitive domains. However, given that research studies cannot assess all real-life listening situations, one cannot conclude that these are the only cognitive domains of relevance, but that they represent those most commonly measured in the context of the limited listening tasks employed in studies, and there may well be other confounding factors such as alternative cognitive strategies that individuals use to compensate (64). The models also highlight that poor fidelity of the input signal (via hearing loss or inappropriate hearing aid processing) can cause greater conflict in resolving speech and, ultimately, a failure to do so. Overall, the models support the finding that speech intelligibility is associated with processing speed, inhibition, WM and long-term storage and retrieval (92) and will consequently decline with age as these narrow cognitive domains deteriorate. It should also be pointed out that a decline in some cognitive domains will not only undermine speech intelligibility, but may also impair the perception of speech quality (113), even where intelligibility is unaffected (114), and undermine an individual’s perception of aided speech.

In summary, certain narrow cognitive domains (Figure 1) will be associated with different types of auditory task. A wide array of cognitive tasks are employed across different studies (92), so the presence and strength of associations between cognition and auditory performance will depend on the narrow cognition domains assessed and the task used to assess them, as well as the type of auditory task, its level of difficulty, the type of stimulus, noise and other cues used (64, 115), the degree of context, vocabulary and visual cues (116–118). Furthermore, there is an association between hearing loss and cognitive decline (119) that may act as a confound in studies. Sensory impairment can affect an individual’s performance on cognitive tests, often requiring recall of spoken words or text-based tests, dependent on hearing and vision, so that it is possible that this causes falsely enhanced associations (61, 120, 121). It may therefore be considered unsurprising that the outcomes of research studies are not wholly consistent because they do not employ consistent paradigms.

## 5. Implications for hearing aid fitting

### 5.1. Principles

The preceding sections have summarized the effects of ageing on peripheral hearing, auditory processing and cognition. It is now important to determine which of these factors are relevant to hearing aid fitting and how some signal processing strategies may create benefit or impediment for individuals with differing degrees of cognition and auditory processing. The “optimum” hearing aid fitting should not solely consider peripheral hearing loss, but should aim to deliver maximum benefit over time, considering hearing loss, auditory processing, cognition and non-auditory factors that affect an individual, their perception of treatment and ability or intention to comply with it. For example, no amount of fine-tuning of a hearing aid’s gain will deliver benefit if there is a perception of unacceptable distortion or if individuals feel unable or unwilling to use their hearing aids. As a result, too many hearing aids remain under-utilized or unused (122). Selection of hearing aid parameters must take all of these factors into account.

Hearing loss degrades the fidelity of the input to the auditory system, undermining its ability to take advantage of temporal and spectral cues and requiring additional effort to resolve mismatches. It is unreasonable to expect that hearing aids can fully restore this input (123). Hearing aids can only address some of the loss in peripheral sensitivity. They cannot directly improve elements of auditory processing or cognition, but they may enhance the signal-to-noise ratio or suppress noise that might distract from the signal or increase listening effort. However, some hearing aid settings may significantly degrade the input signal in ways that hamper auditory processing, undermine speech perception or listening comfort, and increase listening effort (124, 125). The primary role of audiologists should be to address the concerns of, and provide benefit to, the individual patient, so it is important that the patient is given the opportunity for input and that these signal enhancement principles are understood and alternative settings considered (126). In general, hearing aid users with greater cognitive ability appear to benefit more from hearing aids (127). It is therefore important to derive some clear evidence-based principles for hearing aid settings for older adults. Accordingly, for those older adults with reduced auditory processing and cognitive abilities in relevant narrow domains, we can state the following principles based on the preceding review:

1. Older adults will generally be more dependent than younger adults on ENV for speech perception, which is less impaired than TFS with increasing age for monaural listening (46, 47, 128). Older adults will therefore be more susceptible to ENV distortion (124, 129). A primary aim of hearing aid fitting should therefore be to ensure that speech is audible but with minimal distortion to ENV (130).
2. Given that a reduced temporal accuracy with age undermines gap detection (23, 131), quiet gaps between elements of speech should be maintained.
3. Binaural processing of TFS and ENV is degraded with age (46, 47, 52) and impairs interaural level difference (ILD) and interaural time difference (ITD) cues (23, 132). Use of bilateral hearing aids should not further disrupt theses cues, e.g., via fast gain changes that particularly diminish the aided level difference.
4. Reduced binaural processing makes older adults more susceptible to binaural interference (132, 133). There is evidence that some individuals may perform better in noisy situations with a unilateral aid (134) although this is not consistent across studies (135). Unilateral aiding should be considered where there is any indication of binaural interference (136). There should not be a presumption that bilateral aiding is best in every case.
5. General impairment of SIN performance with age (21, 27, 49) indicates a need to improve the signal-to-noise ratio through the use of directionality.
6. Introducing distortion to ENV may increase listening effort and diminish the capacity for listening (100–103). Hearing aid processing should aim to minimize cognitive load and listening effort (86, 96) by avoiding unnecessary distortion to the speech signal, loudness discomfort or other perceptual sound difficulties. Noise reduction settings should be considered (104).

These principles should not be regarded as novel in any way. Directionality has been employed for many years in analogue and digital hearing aids. However, since the inception of digital hearing aids it has been suggested that audiologists should pay greater attention to the distortional aspects of hearing aid processing, rather than solely considering the amplification needed to correct peripheral hearing loss (137, 138). This has also previously been applied specifically to hearing aid fittings for older adults (130). It has long been recognized that hearing aid settings, the associated aided speech recognition and overall satisfaction will be, in part, dependent on cognitive ability (139–141) and that different hearing aid processing strategies will have different advantages and disadvantages for individuals with varying degrees of cognitive function (96). The following sections will consider various hearing aid parameters, how they affect the principles above, and the effect of cognitive change on the fitting process.

Before doing so, it is worth considering more carefully what is meant by “distortion.” In simple terms, distortion can be defined as any non-linear change from the original signal, although the inherent purpose of hearing aids is to modify sound. Multiple signal processing strategies alter speech signals in many ways and it is not a straightforward exercise to measure distortion across its different forms (142), and even less so to equate any measure of disruptive distortion to the perception of a hearing aid user. For example, if one were to measure the distortion introduced by compression, one is immediately faced with the issue of how to combine a measure of change to ENV and a measure of change to TFS, and whether these have detrimental effects after processing in the auditory system. Korhonen et al. (143) suggested a method of assessing modulation in frequency bands, but this only evaluates changes to ENV. Hearing aids introduce multiple sources of distortion and widely-available test box measures, such as total harmonic distortion, are unrepresentative of the overall distorting effects of a hearing aid on the speech signal. The loss of differentiation between the modulation of the speech signal and that of a competing talker or background noise when they are compressed together in a hearing aid is known as “cross-modulation.” This undermines the auditory system’s ability to separate sounds because shared modulation will be interpreted as originating from a single source (144), and can be considered as a loss of information from the original signal. There is more recent evidence that, for complex speech signals, the auditory system uses the interaction between modulations in the frequency and time domains, known as “spectrotemporal modulation,” which is an important determinant in speech intelligibility. Spectrotemporal modulation is undermined by a combination of reduced frequency-tuning and TFS sensitivity (145, 146), although it is unclear how it is distorted by hearing aid processing (147).

There have been numerous initiatives that have attempted to quantify overall distortion and relate it to speech intelligibility and perceived quality; see Kates and Arehart (148) for a summary. Perhaps most promising are the Hearing Aid Speech Perception Index (HASPI) and the Hearing Aid Speech Quality Index (HASQI) (148). These are neural-network models incorporating assumptions of peripheral and central auditory processes, then fit to measurement data for hearing impaired and normal-hearing listeners. The models offer clear differentiation in quality and intelligibility between hearing aids from different manufacturers and between different hearing aid settings (149). It is therefore possible that HASPI and HASQI could become clinical tools that can be used to evaluate distortion. However, to be most useful they would need to be validated against different patient groups because optimal settings are likely dependent on an individual’s cognition or other factors. Moreover, even after validation, there may remain considerable variation between individual preferences. Likewise, it is unclear what trade-off there should be between intelligibility and quality scores, or how this would be determined on an individual basis.

### 5.2. Compression speed

Reducing the dynamic range of sound to match that of a hearing-impaired person, i.e., compression, is a fundamental feature of digital hearing aids. Hearing aids filter sound into frequency bands, or frequency channels, such that different degrees of compression, and other types of processing, can be differently applied within each channel. Wide-dynamic range compression (WDRC) with short time constants, or fast-acting compression (FAC), was employed to enable exaggeration of quieter syllables or phonemes within words by quickly applying greater gain, so is often referred to as “syllabic compression.” This type of compression is based on the fact that some elements of speech may be inaccessible to a hearing-impaired listener, typically high-pitched fricatives for those with a high-frequency hearing loss. FAC aims to restore normal loudness perception across the frequency range (150). Hearing aids with slow-acting compression (SAC) enhance high-frequency sounds solely via the application of different amounts of gain in each frequency channel. In practice, most systems employ similar attack times (1–10 ms), which was to protect patients from sudden loud sounds (150), so “fast” and “slow” hearing aids are largely differentiated by the release times (151). FAC is usually characterized by attack times of 0.5–20 ms and release times of 5–200 ms, whereas the release times for SAC are typically between 500 ms and 2 s (150). The instantaneous gain for any input level and frequency will accordingly be determined by both the gain settings for quiet and loud input levels (i.e., that which should be applied for a long-duration sound) and the compression speed (i.e., whether the aid reacts fast enough to reach the gain setting). It should be recognized that simply comparing fast and slow compression speeds is a simplification. There are various approaches to the implementation of compression speeds in hearing aids that may be applied differently by frequency channel, by direction of change in sound intensity (150), or adaptive systems that change compression speed by acoustic situation (152–157). Furthermore, fast-acting impulse noise protection may be employed as a separate system to WDRC, although the details of these approaches are usually proprietary (158).

**Figure 2:**
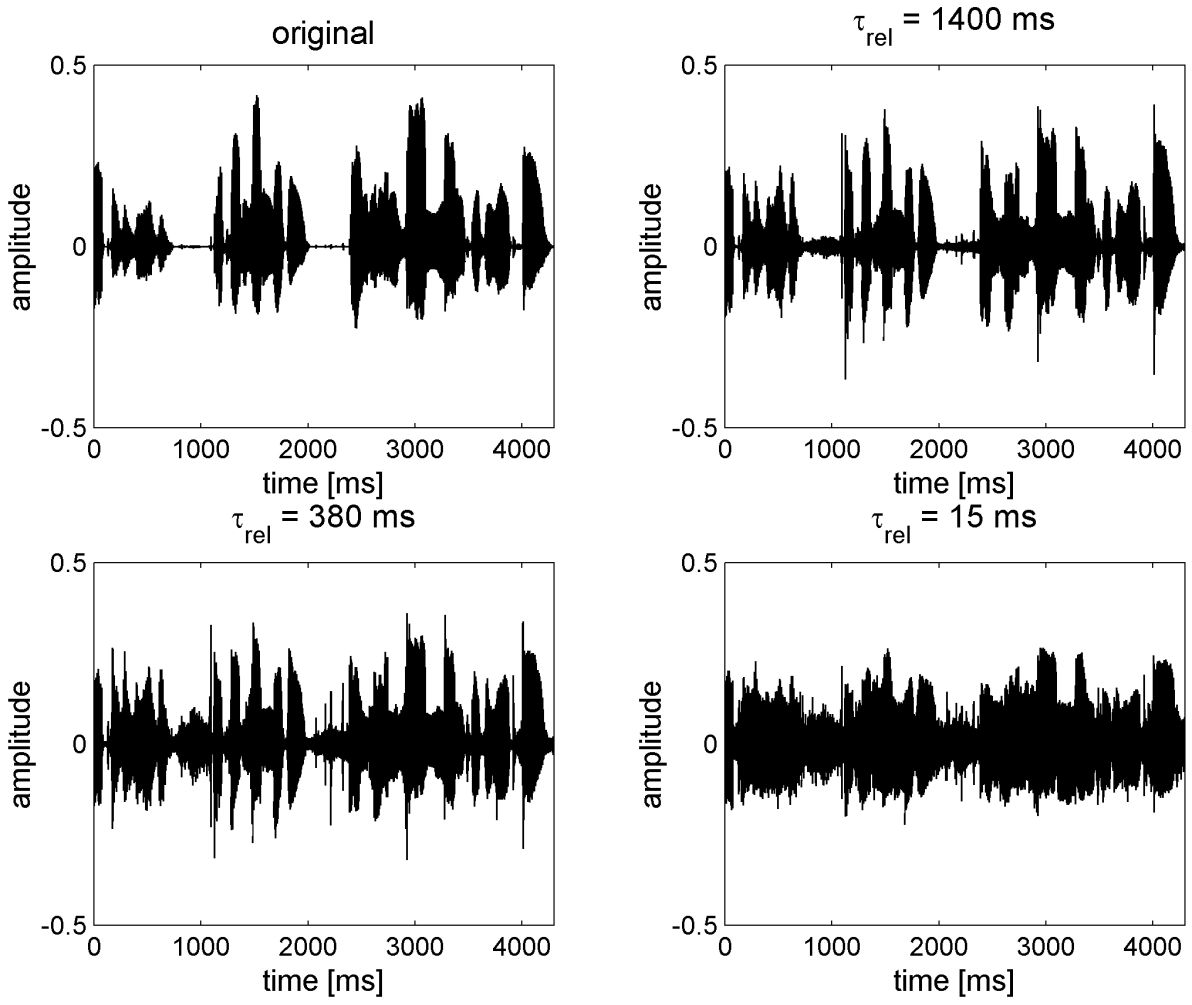
The effect of compression speed on speech envelope cues, reproduced with permission from Holube et al. (159). The original speech signal (top left) is processed through a 16-channel system with an exaggerated compression ratio of 8:1 using various release times (τ_rel_) from long (1,400 ms, slow-acting compression) to short (15 ms, fast-acting compression).

FAC can be interpreted as contravening many of the principles suggested above for individuals with reduced degrees of cognitive function. In general terms, FAC has the effect of “flattening” ENV, whereas ENV is preserved by SAC (Figure 2) (150, 159). FAC may therefore impair speech perception by changing the speech signal in a number of ways: distorting speech envelope cues; amplifying the noise in gaps, undermining gap detection; and reducing modulation detection that enables separation of individual speakers (150, 160–166). FAC might also impair sound localization based on interaural differences, although the evidence for this is weak (167). FAC also impairs localization of sounds in reverberant conditions (168). Even in quiet situations, increasing the number of frequency channels, as is typically deployed in almost all hearing aids, reduces the spectral contrast of vowels and associated speech intelligibility with FAC (159, 169). Proponents of FAC contended that listeners cannot be aware of contrasts in amplitude if a signal is presented below the threshold of the cochlea at a particular frequency (170). However, this argument was largely based on evidence from two-channel systems. In modern multi-channel hearing aids, increasing the number of channels increases the amount of distortion when using FAC (159). Hearing aid users prefer fewer channels when FAC is used, whereas the number of channels does not make a difference with SAC (159). In principle, FAC should only be employed to make weak phonemes audible in quiet conditions for those with mild-to-moderate degrees of hearing loss (171) and does not offer benefit in noisy situations (151, 172). Overall, those with greater degrees of hearing loss tend to benefit less from FAC (173). In truth, to make a proper comparison between FAC and SAC, one should consider the speech signal as presented to the auditory system after processing via the cochlea. As we are unable to do this in humans, we must seek to determine objective or subjective measures of performance from the patient population, such as performance in speech intelligibility tests or self-reported benefit and satisfaction. Other evidence can be sought from animal models in which neural responses can be measured with hearing aid-processed sound (174), suggesting that FAC acts to undermine spectral and temporal contrasts, leading to a failure to restore consonant identification in quiet. Computational models of the auditory system might also be used to assess the effects of different hearing aid processing strategies. These suggest that slow compression leads to a greater restoration of the neural representation of speech than fast compression (175). The HASPI-HASQI model, discussed above, can be employed to compare intelligibility and quality in different scenarios, although it has not been specifically used to compare compression speeds. However, it does suggest that increased processing complexity does not inherently provide better performance (149).

There are a considerable number of studies that compare the outcomes of using hearing aids with FAC and SAC and relate it to individuals’ cognitive scores. A seminal set of studies by Gatehouse et al. (141, 172, 176) compared five linear and non-linear fitting strategies using FAC and SAC, with a crossover design, for older adults with mild-to-moderate sensorineural hearing loss. Three non-linear strategies were employed: slow-slow, using slow-acting release times across low and high frequency bands respectively; fast-slow, a hybrid strategy using fast release times for low frequencies (<1,500 Hz) and slow times for high frequencies; fast-fast, using fast release times in both bands. Speech intelligibility was measured in several conditions, altering the speech presentation level and signal-to-noise ratio. Non-linear hearing aid strategies provided better aided speech recognition than linear approaches, with FAC offering greater benefit than SAC, but the degree of benefit declined with increasing speech presentation level or reduced signal-to-noise ratio. The slow-slow paradigm offered significantly greater listening comfort, and slow-slow and fast-slow were significantly preferred by users. Conversely, fast-fast and fast-slow paradigms had significantly better speech intelligibility, both user-reported and measured. However, whilst the level of a subject’s cognition had no influence on speech tests scores when using SAC (slow-slow or fast-slow), fast-fast compression generated a significant negative correlation between cognition and speech perception, resulting in a wider range of benefit and impediment to patients. In summary, the benefits of FAC over SAC were only accessible to those with better cognitive scores, able to take advantage of increased audibility at the cost of reduced “temporal contrasts,” whereas SAC offered greater benefit than FAC for those with lower cognitive scores. It is notable that these studies used hearing aids with only two channels so, based on the discussion above, one might speculate that the difference observed may have been greater with multichannel hearing aids. The study was replicated with much the same result, further demonstrating that the association between SIN performance and cognitive test scores was stronger when more demanding listening tasks were used (106). Hearing loss was the stronger predictor of SIN performance, relative to WM, when SAC was employed; whereas WM was a stronger predictor than hearing loss when using FAC. Other studies have found WM to predict SIN performance in difficult situations when FAC was applied (177–179), concluding that FAC created a disadvantage relative to SAC for those with lower WM (125). Likewise, stronger preference for SAC relative to FAC, when listening to speech and music, can be associated with individuals with lower TFS sensitivity (180). Hearing aid users with poor TFS sensitivity are also affected more by ENV distortion (129). There is also some interaction between different elements of hearing aid processing. For example, adults may prefer SAC when mild noise reduction is employed, but FAC when strong noise reduction was applied, although the effect sizes were small (181). Conversely, a number of similar studies have found that the relationship between compression speed and SIN performance was not affected by the variation in cognitive scores (177, 182–188). One study suggested that FAC offers greater benefit than SAC in quiet and noisy situations for all users (189), irrespective of cognitive scores, although this showed linear amplification to be better than FAC or SAC, and significant differences were only seen at lower presentation levels. In any case, the researchers went on to suggest adaptive compression speeds that utilized SAC in noisy environments (155).

It is difficult to compare studies because of the different paradigms used for testing speech and cognition, as discussed in Section 4. This is further confounded by variable application of algorithms, the number of channels employed in commercial and research hearing aids, and situations that may not be representative of a user’s daily experience (190). Studies also vary widely in the amount of acclimatization allowed for research participants, if any, and results vary between new and experienced hearing aid users (107, 191). Complete consistency can therefore hardly be expected between studies and a systematic review found it difficult to draw clear lessons (192). On balance, the studies suggest that individuals with lower degrees of cognition will fare worse with FAC, compared to SAC, in some listening situations that are challenging. Those with high cognitive scores will derive benefit from FAC, compared to SAC, in quiet situations. Audiologists must consider audibility, distortion to ENV and listening comfort when setting a hearing aid, amongst other things. Furthermore, the preceding discussion largely addressed only objective benefit, i.e., aided speech intelligibility, in various situations, but not the perceptions of users that will affect outcomes, not least in mediating compliance with treatment. A number of studies show that users generally prefer SAC in noisy, or all, situations (107, 141, 184, 193) or that different individuals have different preferences, with a greater average preference towards SAC (180).

### 5.3. Compression ratio

The compression ratio defines the range of gain applied within a hearing aid channel. A higher compression ratio increases the range of input levels that, after amplification, are audible without being uncomfortably loud and should improve speech intelligibility in quiet, but will reduce intelligibility in background noise (151, 171). The effective compression ratio is dependent on the compression speed. In simple terms, SAC reacts too slowly to reach the highest levels of gain determined by the compression ratio within a short timeframe, such as a word, so the distorting effect on ENV for that word is smaller than FAC (Figure 2), where gain may change within the full range determined by the compression ratio during each word spoken. Consequently, the same compression ratio cannot be set for FAC and SAC systems. A compression ratio of 3.0 or less will have little effect on speech envelope cues for SAC (194). However, the amount of distortion introduced by FAC will be broadly proportional to the compression ratio and it is generally recommended that it is not set greater than 3.0 (195) or 5.0 (151, 152). Distortion will be further exaggerated by increasing the number of frequency channels (159). Increasing the compression ratio can reduce consonant recognition (196) and overall speech recognition (197). When asked to subjectively rate the quality of speech, listeners tend to prefer lower compression ratios in quiet and even more so (CR ≤ 2.0) in noise (159, 197, 198). It should also be noted that that the compression ratio is measured with steady-state signals and the effective compression for a fluctuating signal like speech is lower (199); hence, the longer the release time, the lower the effective compression ratio (151, 152). A greater compression ratio can therefore be set for SAC without noticeable distortion, and the additional comfort availed can be interpreted as a benefit of SAC (150). In order to avoid distortion to ENV for older adults, compression ratio must be reduced significantly if FAC is used.

### 5.4. Noise reduction

Noise reduction algorithms aim to reduce the level of non-speech sounds and increase comfort. A meta-analysis found that noise reduction does not consistently improve speech intelligibility, but is moderately beneficial for sound quality and comfort (200). Noise reduction can be fast or slow-acting and can cause disruption to the speech signal by alterations to gain in specific frequency channels, and this may be differently tolerated by those with different degrees of cognition. As noted above, the strength of noise reduction employed interacts with the preference for FAC or SAC (181). Nevertheless, those with relatively good cognitive scores benefit from stronger noise reduction, whereas it may be detrimental to those with lower scores (125). Background noise impairs WM function, making it harder to recall words, especially for more complex listening challenges. Noise reduction may overcome this problem by reducing the demands on WM, but may mainly apply to those with higher degrees of WM in more challenging tasks (201). Some studies suggest that it may apply to those with lower degrees of WM only in less challenging tasks (202, 203). Strong noise reduction likely impairs speech intelligibility for those with low WM or executive function (204, 205), although moderate noise reduction is preferred in most situations and some users prefer strong noise reduction despite the loss in intelligibility (206). Overall, moderate noise reduction likely improves listening comfort without undermining speech intelligibility in most cases, although there is some variability in individual preference.

### 5.5. Other hearing aid parameters

Hearing aids, by their nature, alter a sound signal and inherently introduce some form of distortion relative to the original. Other features manipulate the sound, including frequency-dependent gain, directional microphones, feedback management, frequency lowering, expansion and wind-noise reduction. Any hearing aid processing that distorts ENV may impair speech perception and this may be especially detrimental to those with lower degrees of cognition (207). However, there is less evidence relating the benefits or drawbacks of these features in relation to cognition.

There is some evidence that frequency-lowering, or frequency compression, can benefit those with higher WM scores but it might act to undermine intelligibility in those with lower WM scores (124). A meta-analysis of frequency-lowering suggested that it has a small benefit in quiet situations, although results were inconsistent and situation-dependent (208), and it may impair speech perception in noise (209). However, frequency lowering might be beneficial in noise for younger adults or those with steeply-sloping hearing loss (103). Frequency-lowering might be trialed as an option, but may impair listening for those with lower cognitive scores.

One dimension of hearing aid setting that has well-established benefits and consistently positive outcomes is the use of directionality to improve signal-to-noise ratio (206) so it is not considered in any detail here. Whilst directionality applies gain preferentially to sounds based on direction, it does not inherently distort the sound in the same way as non-linear processing schemes. There is a similar benefit from directionality for any compression speed irrespective of the degree of WM function (210).

### 5.6. Non-auditory factors

This review is focused on hearing aid parameters and their effect on aided outcomes for older adults with cognitive change related to normal ageing. Whilst not a subject of this review, we should be cognizant of the fact that hearing care for older adults must include a wider appreciation of their needs including physical and listening comfort, psychological, social, behavioral and environmental considerations, and these have implications on hearing aid settings, acceptance and outcomes (211). For example, despite a declining ability to make use of multi-sensory inputs (212), older adults still benefit from visual inputs for speech perception (118) so visual deficits should be corrected. Education of families and careers should incorporate some understanding of auditory decline and enable them to make adjustments, e.g., via avoidance of fast speech (212), use of clear speech, providing cues before speaking (109), maximizing context (213), familiarity (214) and environmental adjustments to reduce background noise. Other non-auditory factors associated with the comorbidities of age and reduced cognition that can inhibit successful use of hearing aids include dexterity, self-efficacy, attitude, motivation, family support and self-image (98, 99, 215, 216) as well as the “simple” or “complex” manner in which a hearing aid is configured (e.g., with or without programs or control buttons). The cognitive status of individuals will also affect clinical interactions because higher degrees of fluid intelligence enable them to engage more with life (217), handle problems such as acclimatizing to a hearing aid, and to be more proactive in taking action to address their health (218). In short, a hearing aid fitting must provide the patient with a solution that they find manageable and acceptable and, as seen in much of the research discussed above, patients’ preferences are not always aligned with the solution that gives maximum speech intelligibility. In summary, whilst we attempt to determine the best approach to setting hearing aid parameters in this review, we recognize that these recommendations should be interpreted within the broader scope of an individual’s needs.

## 6. Discussion: a pragmatic approach to setting hearing aids for older adults

The preceding sections of this paper have reviewed the literature to determine how age-related changes to cognitive domains and auditory processing alter speech perception and, in particular, an individual’s relative dependence on different speech cues that might be disrupted by different approaches to hearing aid processing. In this section, we aim to apply the evidence to the clinical hearing aid fitting process in order to provide pragmatic guidance to clinicians.

As part of a patient-centered care model (219) the audiologist’s primary role is to offer support and provide benefit to each individual, where the total benefit can be considered as a combination of objective and perceived benefit, preference, and ability to comply with treatment. This perspective means that the “optimal hearing aid fitting” must therefore address all of these aspects of care. In consequence, audiologists require a broad skill set that includes counselling (219) and some level of technical understanding. However, some relevant technical factors, not least compression speed, are rarely provided by hearing aid manufacturers in specification sheets, nor is it easy in our experience for audiologists to acquire such information. This is a fundamental concern because compression speed and compression ratio are primary determinants for the amount of distortion introduced to ENV. Given that audiologists are, in part, dealing with the hearing aid as a “black box,” and that research findings are not always consistent, it is perhaps unsurprising that there are no clear technical guidelines for fitting hearing aids to older adults (220).

However, the research is broadly consistent in a number of ways. First, older adults are more likely dependent on ENV for understanding speech. FAC causes distortion to ENV that can undermine speech perception, whilst emphasizing elements of TFS that cannot be utilized by many older hearing aid users, and might disrupt binaural inputs. Second, hearing aid users tend to prefer SAC on average, although individuals have different preferences. FAC is likely better in quiet for those with good cognitive processes related to hearing, but likely degrades speech intelligibility and the acceptability of hearing aids for those with lower degrees of cognition. Third, the variation in cognitive processes between individuals increases with age, so we cannot know which specific settings offer the most benefit for an individual, nor whether objective benefit will be aligned with their preference.

The combination of peripheral and central auditory decline therefore means that older adults represent some of the most complex patients, as well as the most numerous. Consequently, age-related hearing loss should never be treated as “standard care” simply because of its high prevalence. Determining the correct hearing aid strategy for an individual hearing aid user is therefore a major challenge. One might think that it would be useful to undertake tests of cognition or TFS sensitivity in clinic. However, it is uncertain that a test score could usefully indicate a specific course of action, nor which cognitive tests should be used. Verbally-delivered cognitive tests are affected by hearing loss and there is no well-established test that has been shown to be unaffected by sensory impairment (61, 120, 221). It is unclear whether audiologists are able to conduct cognitive tests sufficiently well and their utility in clinical situations needs evidencing (222). Speech testing might give some indication of overall ability and it has been suggested that it be more widely used (223) because standard audiometry provides no useful evidence regarding auditory processing and cognition. However, it is equally unlikely that a speech test score could equate directly to a specific hearing aid setting and user preference. It is also unclear whether clinical speech-testing is sufficiently sensitive to determine any benefit derived from alternative hearing aid settings. Consequently, in addition to the current lack of applicable tools, it is debatable whether the additional loading on clinical time would yield sufficient benefit to be justified. In any case, many audiology services face demand and resource pressures, particularly in public sector systems with universal treatment, so extending the test battery significantly may not be implementable.

We therefore propose a pragmatic approach to hearing aid setting that could be implemented within typical current fitting appointments. The principles above can be employed to ensure that relevant factors are considered for older adults, which we define nominally as those over 55 years of age in line with the English NHS definition of age-related hearing loss. We believe the following statements concerning hearing aid settings for older adults follow directly from the evidence:

1. All factors that introduce distortion to ENV should be explicitly considered in hearing aid fittings. This should primarily include compression speed and compression ratio, but also noise reduction, frequency lowering and other advanced features.
2. Compression speed and compression ratio are primary determinants of the degree of distortion introduced to ENV. This indicates a need for hearing aid manufacturers to publish compression speeds as part of their standard product information, and provide products that allow clinicians to change compression speed, or offer adaptive compression speed algorithms that can be selected appropriately for each individual.
3. SAC should be considered as the default setting for older adults and should be employed in noisy environments for all hearing aid users. However, recognizing the variation in preference between individuals, both SAC and FAC approaches can be provided in separate hearing aid programs and the audiologist can employ validation techniques to assess which is best set as the default program. There is no good reason that hearing aids should default to FAC for older adults.
4. If FAC is used, the compression ratio should be reduced to levels that avoid ENV distortion. There is no clear evidence that suggests a specific value, so it is difficult to provide robust guidance. Our own clinical experience and discussions with hearing aid manufacturers suggests a value as low as 1.5. This is, admittedly, anecdotal but there is some evidence to suggest that linear aids are preferred over WDRC aids with FAC and a compression ratio of 2.0 when subjectively rating the quality of aided speech (159). Users also prefer CR ≤ 2.0 in noise (197, 198).
5. Directionality is always beneficial to improve the signal-to-noise ratio. This might also include a consideration of ear-molds in place of open-fittings, because the latter reduces directional benefit (224, 225). Alternatively, other assistive devices and accessories may be considered to improve directionality or enhance the signal-to-noise ratio.
6. Noise reduction is likely best set to moderate, although this depends on each hearing aid manufacturer’s approach and the evidence base is not strong. Strong noise reduction may well impair speech perception, especially for those with lower degrees of cognition. However, the user may be offered options or multiple programs because some may prefer stronger noise reduction in some situations.
7. Other advanced hearing aid features, such as frequency lowering, may also distort the signal in ways that further impair speech intelligibility for those with reduced abilities in some cognitive domains. The evidence is generally weak, so these should be considered as options for each individual.
8. Unilateral aiding should be considered where binaural interference is suspected. This is easily achieved in clinic by comparisons of speech intelligibility and listening comfort with bilateral hearing aids versus the left and right hearing aid working unilaterally. In some cases, clinicians may find that the individual’s speech intelligibility is significantly better when aiding only one of the ears, and intelligibility may be impaired or the sound distorted whenever gain is applied to the other ear.

One should recognize that any recommendation regarding hearing aid parameters cannot be prescriptive given the current state of knowledge and considering the wide variability in individual needs. This implies that there must be some process of ensuring that hearing aid settings are optimal, or at least acceptable, for each individual. Overall, we believe that a full consideration of hearing aid parameters needs to be combined with the over-riding principles of patient centered care. This engenders a need to balance the key elements of the hearing aid fitting process, including verification, validation and counselling. “Verification” is the process of matching hearing aid gain to a prescribed target using real ear measurements (REMs). “Validation” should aim to provide evidence that all hearing aid settings are suitable for an individual. This may include simple tests of loudness discomfort and speech intelligibility, more advanced techniques such as speech mapping, paired comparison approaches (226), or questionnaires and self-reporting tools (227). Finally, “counselling” addresses non-audiometric factors such as the patient’s expectations, self-image and self-efficacy (228). This approach will likely improve the rate of treatment compliance (229, 230). The following statements are not novel, but we believe they are worth re-stating in light of the principles discussed above for setting hearing aids for older adults:

9. Conducting verification and matching gain to a prescription target does not, in itself, make a good hearing aid fitting. The verification process is solely aimed at setting individualized hearing aid gain and does not encourage a consideration of the other parameters highlighted in this review. For example, setting the gain to a prescription target for 50, 65, and 80 dB input levels may lead to high compression ratios where FAC is used, causing inappropriate ENV distortion. Verification is an important step that may offer a large (231) or small (232) benefit, but the resulting gain settings must be considered in the light of the other parameters discussed above and adjusted appropriately.
10. The only clinical process that can evaluate overall hearing aid settings and their suitability for an individual is validation (233). Accordingly, we believe that it is important that verification is always followed by proper validation in clinic (234) and that clinicians should consider the balance of time given to each process during fitting appointments. The hearing aid user should not perceive distortion and loudness discomfort, and sufficient speech intelligibility and sound acceptability should be demonstrated. In short, clinicians should consider both the intelligibility and quality of speech (235). However, it is recognized that current validation techniques, e.g., using live voice or speech mapping, are subjective and more objective methods of assessing the likely perception of overall distortion would be helpful. Appropriate objective methods are currently lacking in clinical practice.
11. Hearing aids cannot overcome all of the peripheral and central auditory deficits discussed in this review, so wider considerations should be addressed to achieve the optimum outcome in the perception of the individual user. Audiologists must, of course, consider other non-auditory factors. Informational counselling regarding central auditory decline should aim to set reasonable expectations for the hearing aid user and their families or careers (236, 237) and can promote modification of the environment and behaviors related to the elements of decline in central auditory processing discussed above. This may include use of clear speech, visibility of mouth movements and reducing background noise.

## 7. Implications for further research

Research studies use a wide array of both cognitive and listening tasks, so the associations observed between them are variable. Although WM tasks predominate in many studies, it is not the only domain of cognition that is relevant (92) and we are yet to fully understand which elements of cognition might relate to every listening condition (64). It would seem prudent to consolidate a range of cognitive tests in research studies. There is also a lack of validated cognitive tests for those with a sensory impairment, because many tasks are delivered verbally or visually, so hearing loss may be a confounding factor (61, 120, 121) and must be considered as part of research design. In large part, studies relate cognitive status to performance in listening tasks, although some studies relate it to user preference. It should be clear that individual preferences are not always aligned to objective performance and may be based more on listening comfort, effort or some other perception of quality, so it would seem sensible to measure both objective performance and subjective benefit in research studies.

Whilst there is variation in the evidence, it appears to us that the balance of evidence suggests that slow-acting compression may be the best default position for most older adults along with other cautious settings for compression ratios and noise reduction, although accepting there will be individual variations. However, further research should seek to confirm the size of the effect. Furthermore, most research on the effect of hearing aid settings considers one parameter in isolation whilst other parameters are held constant. This is unlikely to reflect the overall function of commercial hearing aids and assess the interaction between different hearing aid features. Recent work has demonstrated the interactions between compression speed and other settings, such as noise reduction and directionality (181, 210), so further research should establish whether combinations of hearing aid processing strategies negate the overall effect of individual settings in the user’s perception. It is also important to be able to evaluate balanced measures of speech intelligibility and quality, and relate them to the overall perception of distortion. This indicates a need to further develop clinical tools, such as HASQI and HASPI (148, 149), to determine an objective measure of distortion appropriate for individuals with varying degrees of hearing loss and cognitive ability, that also aligns with user perception. This further begs the question of whether some test of cognitive ability is useful or implementable within clinical practice.

In this paper we have proposed an approach to setting hearing aids for older adults. Whilst this may be derived from the balance of evidence, it is not specifically validated. We have therefore designed a randomized control trial based on the principles discussed in this paper, pre-registered on the Center for Open Science’s Open Science Framework (OSF), at https://osf.io/fdzeh. The SHAOA (Setting Hearing Aids for Older Adults) study has been approved by the UK Health Research Authority and National Health Service (NHS) Research Ethics Committee (IRAS ID 313159), and it has been adopted onto the NHS National Institute for Health Research (NIHR) portfolio. Finally, in suggesting this approach, we make an implicit assumption that audiologists may not consider all of the factors that have been highlighted here, which we have not evidenced. Accordingly, we will also complete an online survey of UK clinicians to evaluate this, under University of Manchester ethics approval.

## Author contributions

RW conducted an initial long-form literature review which was reviewed with comments and changes from HD and AH. RW wrote the draft of this paper with comments and revisions from HD and AH. RW is Principal Investigator for ongoing research with HD and AH as academic supervisors. All authors contributed to the article and approved the submitted version.

## Funding

RW is funded by the NHS National School of Healthcare Science (NSHCS) as part of the NHS Higher Scientific Specialist Training (HSST) scheme, supporting work at the Royal Berkshire NHS Foundation Trust. HD and AH are supported by the NHS National Institute for Health Research (NIHR) Manchester Biomedical Research Centre.

## Conflict of interest

The authors declare that the research was conducted in the absence of any commercial or financial relationships that could be construed as a potential conflict of interest.

## Publisher’s note

All claims expressed in this article are solely those of the authors and do not necessarily represent those of their affiliated organizations, or those of the publisher, the editors and the reviewers. Any product that may be evaluated in this article, or claim that may be made by its manufacturer, is not guaranteed or endorsed by the publisher.
