# Erratum: A review of auditory processing and cognitive change during normal ageing, and the implications for setting hearing aids for older adults

**DOI:** 10.3389/fneur.2023.1254802

**Published:** 2023-07-18

**Authors:** Frontiers Production Office

**Affiliations:** Frontiers Media SA, Lausanne, Switzerland

**Keywords:** ageing, cognitive performance, hearing aids, auditory processing, compression speed, compression ratio, noise reduction

Due to a production error, a correction has been made to a sentence in the section “The effects of ageing on auditory processing”, paragraph number 3. The paragraph now reads:

“Temporal information in speech cues can be differentiated across a number of frequency bands (29): information about the speech envelope (ENV) is carried primarily in the frequency band below 50 Hz; information about voicing and periodicity primarily in the frequency band between 50 and 500 Hz; and information about temporal fine structure (TFS) in the frequency band above 500 Hz. ENV and TFS are the most important cues forming the basis of speech perception, frequency perception and localization (30). However, for those with sufficient hearing, ENV and TFS provide redundant cues that support processing of the speech signal (31). A difference in the pitch of voicing helps differentiate individuals in multi-talker situations (32). Vocoder experiments that replaced TFS with a carrier signal or noise, which was modulated by ENV, demonstrated that only a small number of frequency bands are required for successful speech perception in quiet even when TFS was lacking (33). However, the same was not true when speech was presented in a noisy background. Adding back TFS to a signal progressively improved SIN performance (34, 35). The ability to make use of ENV with little TFS can also be evidenced by the success of cochlear implants, which provide a grossly reduced representation of spectral detail (29, 36). Besides supporting speech perception in noise (36–38), TFS also supports binaural localization and separation of competing sounds (39), perception of pitch and music (29, 30) and perception of motion (40). Individuals with peripheral hearing impairment cannot make good use of TFS which can lead to difficulties hearing in noisy situations (41).”

The publisher apologizes for this mistake. The original article has been updated.

